# An Open‐Source Multifunctional Testing Platform for Optical Phase Change Materials

**DOI:** 10.1002/smsc.202300098

**Published:** 2023-11-20

**Authors:** Cosmin-Constantin Popescu, Khoi Phuong Dao, Luigi Ranno, Brian Mills, Louis Martin-Monier, Yifei Zhang, David Bono, Brian Neltner, Tian Gu, Juejun Hu, Kiumars Aryana, William M. Humphreys, Hyun Jung Kim, Steven Vitale, Paul Miller, Christopher Roberts, Sarah Geiger, Dennis Callahan, Michael Moebius, Myungkoo Kang, Kathleen A. Richardson, Carlos A. Ríos Ocampo

**Affiliations:** ^1^ Department of Materials Science & Engineering Massachusetts Institute of Technology Cambridge MA 02139 USA; ^2^ NASA Langley Research Center Hampton VA 23681 USA; ^3^ Advanced Materials and Microsystems Group MIT Lincoln Laboratory Lexington MA 02421 USA; ^4^ The Charles Stark Draper Laboratory, Inc. Cambridge MA 02139 USA; ^5^ CREOL The College of Optics & Photonics University of Central Florida Orlando FL 32816 USA; ^6^ Department of Materials Science & Engineering University of Maryland College Park MD 20724 USA; ^7^ Institute for Research in Electronics & Applied Physics University of Maryland College Park MD 20742 USA

**Keywords:** in situ characterization, optical properties, phase change materials, phase transitions, photonic devices

## Abstract

Owing to their unique tunable optical properties, chalcogenide phase change materials are increasingly being investigated for optics and photonics applications. However, in situ characterization of their phase transition characteristics is a capability that remains inaccessible to many researchers. Herein, a multifunctional silicon microheater platform capable of in situ measurement of structural, kinetic, optical, and thermal properties of these materials is introduced. The platform can be fabricated leveraging industry‐standard silicon foundry manufacturing processes. This platform is fully open‐sourced, including complete hardware design and associated software codes.

## Graphical abstract

Chalcogenide phase change materials show great promise for tunable nanophotonic devices. An open‐source electrical switching platform enabling in situ, high‐throughput, and multimodal testing of phase change materials is presented. The platform will accelerate the discovery and characterization of new phase change materials and facilitate their integration with on‐chip photonic devices.

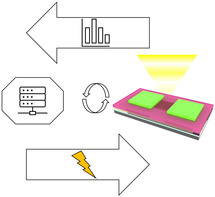

## 1 Introduction

Phase change materials (PCMs) are a class of chalcogenides featuring a large change in electrical and optical properties between their amorphous and crystalline phases. For decades, these materials have been used in optical data storage as well as electronic memories with exemplary compositions such as Ge_2_Sb_2_Te_5_ (GST) and Ag_*x*_In_*y*_Sb_2_Te (AIST).^[^ ^1^, ^2^ ^]^ In the past decade, they have been gaining increasing attention due to their potential uses in photonics.^[^ ^3^, ^4^, ^5^, ^6^, ^7^, ^8^, ^9^ ^]^ The key trait that underpins this emerging field is the nonvolatile, large optical property contrast between their amorphous and crystalline states. This trait foresees a cohort of energy‐efficient, ultra‐compact reconfigurable photonic devices for applications such as neuromorphic optical computing,^[^ ^10^, ^11^, ^12^, ^13^, ^14^ ^]^ photonic memories,^[^ ^15^, ^16^, ^17^, ^18^ ^]^ active metasurfaces,^[^ ^19^, ^20^, ^21^, ^22^, ^23^, ^24^, ^25^ ^]^ tunable filters,^[^ ^26^, ^27^, ^28^ ^]^ reflective displays,^[^ ^29^, ^30^, ^31^, ^32^ ^]^ thermal camouflage,^[^ ^33^, ^34^, ^35^ ^]^ programmable photonic circuits,^[^ ^36^, ^37^, ^38^, ^39^, ^40^ ^]^ and more.^[^ ^41^, ^42^, ^43^ ^]^

Despite the surge of interest in their optical applications, the characterization of PCMs in environments relevant to photonic device settings, quite distinct from electronic memory configurations, is scarce. In photonic devices, electrothermal switching using resistive microheaters, which avoids nonuniform crystallization due to filamentation,^[^ ^44^ ^]^ is the preferred approach for controlling the phase structure in PCMs. Although a number of microheater platforms made of metals,^[^ ^45^, ^46^ ^]^ transparent conducting oxides,^[^ ^47^, ^48^ ^]^ doped Si,^[^ ^40^, ^49^, ^50^ ^]^ and graphene^[^ ^51^, ^52^, ^53^ ^]^ have been demonstrated for this purpose, the phase transition characteristics of PCMs and their corresponding structural and thermal properties have not been systematically investigated. In addition, the failure mechanisms of PCMs in the context of photonic applications, which dictate their reliability and cycling endurance, have not been studied.^[^ ^54^ ^]^ Moreover, photonic applications also stipulate performance requirements distinct from those in electronic memories, such as low optical attenuation and, in some cases, moderate crystallization speed.^[^ ^44^ ^]^ Therefore, a series of new PCM compositions tailored for photonic applications exemplified by Ge_2_Sb_2_Se_4_Te (GSST),^[^ ^55^, ^56^, ^57^ ^]^ Sb_2_S_3_,^[^ ^58^ ^]^ Sb_2_Se_3_,^[^ ^59^ ^]^ Ge_2_Sb_2_Te_3_S_2_,^[^ ^60^ ^]^ and In_3_SbTe_2_^[^ ^61^, ^62^ ^]^ have been developed in recent years. Compared to the archetypal GST family, the structural, kinetic, thermal, and cycling characteristics of these emerging optical PCMs are much less understood. A platform that enables comprehensive characterization of PCMs in environments pertinent to photonic device applications is thus desired.

Herein, we detail the design and implementation of a multifunctional platform for optical PCM characterization. The platform uses doped silicon‐on‐insulator (SOI) microheaters to actuate phase transition in PCMs. We choose SOI heaters because they form the backbone of most photonic integrated circuits, their fabrication is broadly accessible to the community through commercial photonic foundries, their infrared transparency allows transmissive optical interrogation, and finally, they can further act as a probe for local temperature measurement via Raman thermometry. As we will show later, the platform integrates multiple in situ characterization methods to reveal a rich wealth of information regarding the structural, kinetic, optical, and thermal properties of PCMs. Finally, we foresee that the platform, which can be readily coupled with combinatorial deposition,^[^ ^63^, ^64^ ^]^ can also facilitate high‐throughput screening of PCMs to expedite new PCM discovery.

## 2 Testing Platform Setup

**Figure 1:**
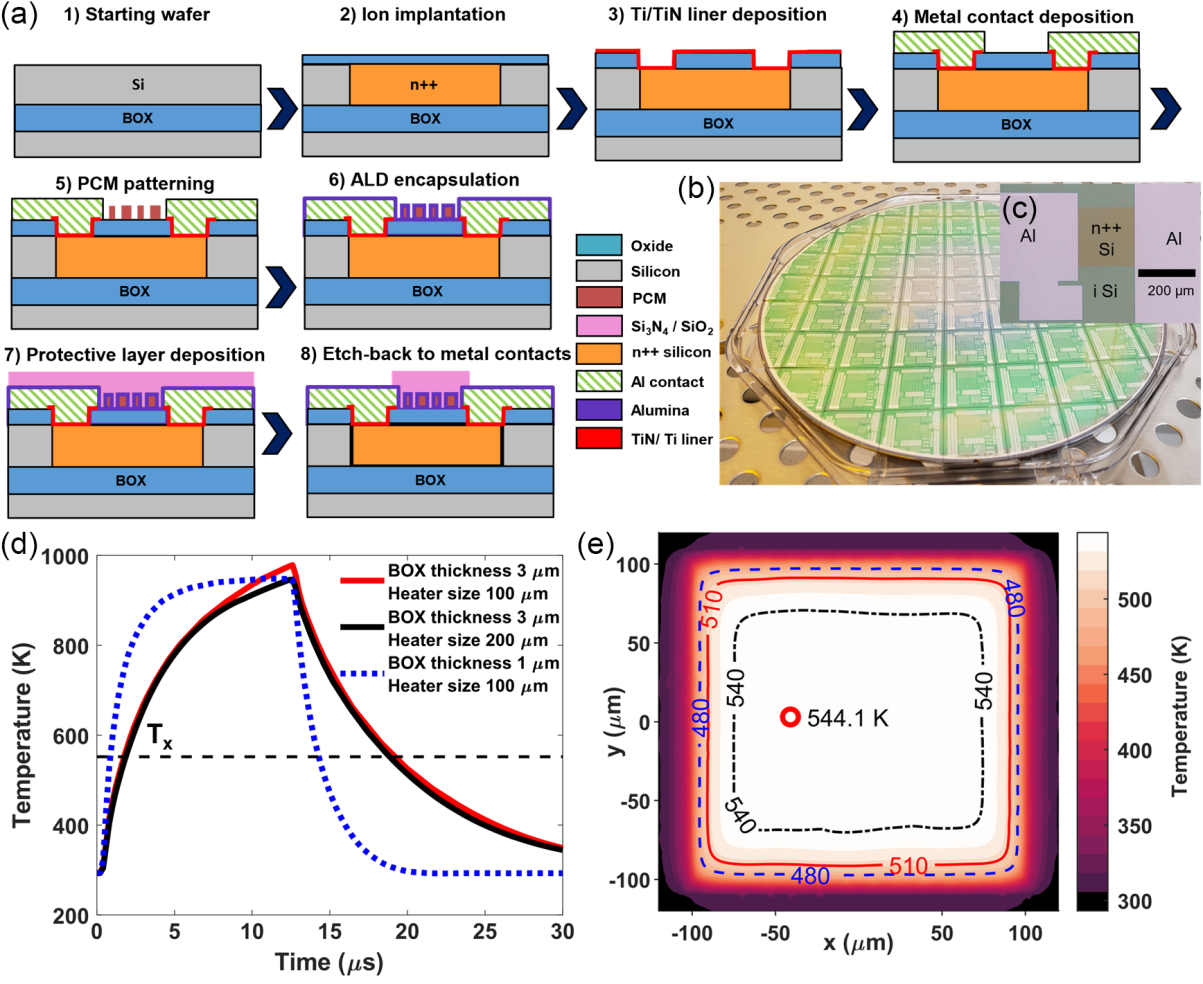
a) Fabrication sequence for the PCM‐integrated SOI heaters. b) Photo of a 200 mm wafer with the heater arrays. c) Top‐view optical microscopy image of a microheater. d) Simulated transient temperature responses at the center of the heater for 3 and 1 μm buried oxide for 12 μs amorphization pulses of 17 V (solid red), 32 V (solid black), and 24 V (dashed blue). e) Transverse, steady‐state, temperature distribution across a 200 μm microheater resulting from a DC voltage, being applicable for crystallization. The red circle highlights the point of maximum temperature, with its position being influenced by the meshing of the heater in an area with low local temperature variation.

The SOI microheaters are fabricated at the MIT Lincoln Laboratory Microelectronics Laboratory. We note that similar fabrication processes are also available through standard multiproject wafer shuttle runs in most photonic foundries (e.g., the base active photonic integrated circuit (PIC) process provided by AIM Photonics). A schematic of the fabrication sequence is illustrated in **Figure** **1**a. Wafers with a 150 nm SOI layer and 1 μm buried oxide were used as the starting substrates. The microheaters were n‐doped with implanted phosphorous ions with 80 kV and a dose of 10^16^ cm^−2^ followed by rapid thermal annealing for 10 s at 1000 °C (step 2 in Figure 1a). 10 nm of SiO_2_ was grown on the device and an etch to the doped Si regions was performed. An adhesion layer of 10 nm Ti and 20 nm TiN was deposited on the exposed Si, followed by an etch back to define the contact regions (step 3). 350 nm of Al was deposited as the contacts (step 4). When needed, the chips were backside polished to facilitate transmissive optical measurements. Prior to PCM deposition, the chip was patterned with photolithography using 2 μm AZ nLOF 2020 resist and developed with AZ 300 MIF developer. The PCM was then deposited via thermal evaporation following established protocols,^[^ ^56^ ^]^ followed by pattern lift‐off in acetone (step 5). The PCM films can be lithographically patterned to introduce optical functions or to mitigate morphology‐dependent failure mechanisms (e.g., liquid‐phase morphological instability or dewetting). The PCM was subsequently capped via atomic layer deposition in 20 nm Al_2_O_3_ (at 110 °C in a Unitronics ALD system) and then further encapsulated in a thick reactive sputtered SiN_*x*_ (deposited using Si targets with gas flow rates N_2_:Ar 6:6 sccm at 3 mTorr pressure on an AJA Orion 5 system) protective layer (steps 6 and 7). The deposited encapsulation layers were removed from above the metal contact pads using reactive ion etching, specifically SF_6_ and Ar plasma with a hard‐baked AZ 3312 resist mask. After the etch‐back, the remaining resist was removed via O_2_ plasma. The microheaters are connected to a printed circuit board via wire bonding using an MEI model 1204 D ball bonder (step 8) to complete the microheater fabrication and packaging processes. It is preferable but not necessary that the PCB contact pads used for wire bonding are of the same metal as the connecting wire (i.e., Au–Au or Al–Al) for easier bonding. The typical resistance at 1 V was around 44–46 Ω but at higher voltage, it would increase as result of temperature rise of the heater. For square heaters that also means that the sheet resistance was around 44–46 Ω □^−1^. For a 44 Ω heater, ignoring series resistance contributions, it implies that the resistivity of the doped Si used was around 6.6 × 10^−6^ Ωm or 1.5 × 10^5^ S m^−1^ conductivity. For 150 μm heaters using typically 35 V pulses with 13 μs time width, a rough estimation of $V^{2}/R=27.8\;W$ of power is generated, meaning the areal power density is 1.24 GW m^−2^ and the volumetric power density can be estimated around 1.15 PW m^−3^. The heat was assumed to be uniformly dispersed across 150 nm of doped Si, 180 nm of GSST, and 740 nm of SiN_*x*_. The total energy density dissipated is estimated at 15 GJ m^−3^. These values are only rough estimations and serve only as starting points for other researchers. Thicker cladding or PCM layers or larger heaters will require higher voltages. Smaller heaters, thinner PCM and cladding layers, and thicker SiO_2_ layers will result in less heating and less voltage required. These pulse and power density parameters are similar to the ones in the work by Aryana et al.^[^ ^65^ ^]^ Specifically, at 45.5 V and 40 Ω for a 200 μm × 200 μm heater, the areal power density is 1.29 GW m^−2^, similar to the power density above. Nonidealities such as resistivity increase at higher temperatures and variations in the total thickness/mass of material heated may explain the departure from simulated values.

**Figure 2:**
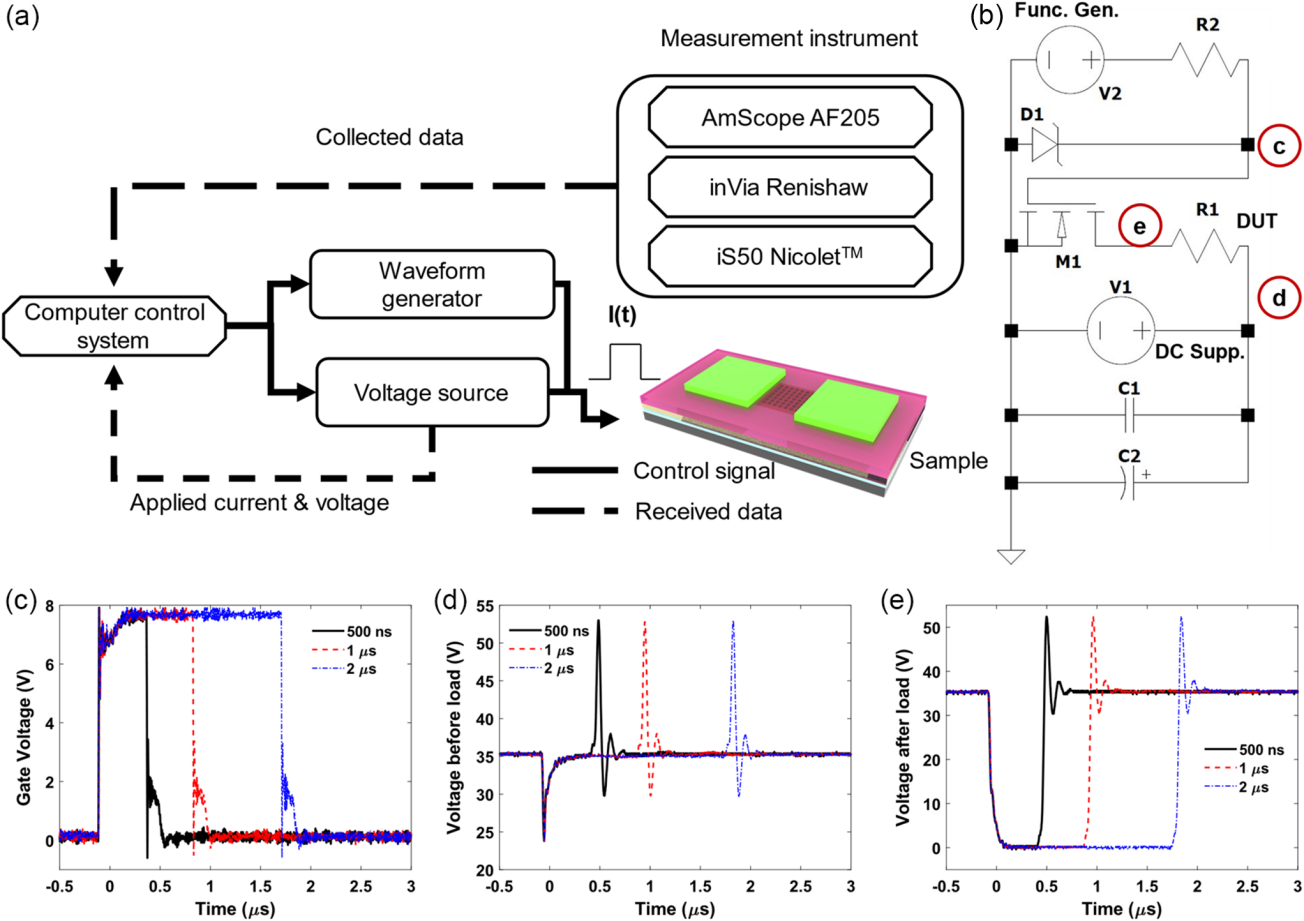
a) Block diagram of the multifunctional characterization platform. b) Electric circuit diagram for high voltage pulse generation. c–e) Voltage measurements from an oscilloscope across the load measured at the transistor gate; d) before the load; and e) after the load.

**Figure** **2** shows a schematic of the experimental setup. In particular, Figure 2b shows the electronic circuit for pulse amplification, where the microheater is denoted as *R*_1_. An IRF 510 power MOSFET transistor (*M*_1_ in Figure 2b) is used as a switch, and two bypass capacitors (*C*_1_ and *C*_2_ in Figure 2b) (a ceramic 1 μF capacitor and an electrolytic 470 μF capacitor for 50 V, respectively) are connected from the power supply to ground to supply the pulse of heater current with only a small drop in the power supply voltage at the source. A 1N4745A Zener diode (*D*_1_) and 100 Ω resistor (*R*_2_) are connected as shown in the diagram at the gate of the transistor. The diode protects against voltages above 16 V from being applied to the gate while the series resistor *R*_2_ limits voltage gain at high oscillation frequencies. These are connected and encased in a Pomona Electronics 3234 shielded box with 4 BNC connectors. A power supply Keithely 2200‐60‐2 is used as DC voltage source (V1) and a function generator Agilent 33 250 A (V2) is used to apply voltage pulses at the transistor gate with a rise/fall time of 5 ns. The communication to the power supply was done via USB to RS‐232 adapter cable with a null modem connection RS‐232 cable in series (for more details check the handshake selection for RS232 in the manual). Both instruments received commands via SCPI code which can be found in their respective manuals.^[^ ^66^, ^67^ ^]^ The waveform data collection was performed using a DPO 2014 Tektronix oscilloscope. The system is controlled via a MATLAB script (available through Github^[^ ^68^ ^]^) which allows synchronization with other instruments. During continuous cycling tests, a delay time between pulses (up to 30 s) is applied to allow for complete heat removal from the regions surrounding the microheaters. This prevents overheating of the devices and damage to the metal contacts.

Figure 2c–e shows exemplary measured voltage waveforms at three different points in the circuit (labeled in Figure 2b). Ringing can be observed at ramp‐up and ramp‐down of the voltage pulses, although its impact on the heater temperature transient is negligible provided that the ringing oscillation period is considerably smaller than the thermal time constant of the heater. It is noted that the function generator is specified for a 50 Ω load at the output port following a 50 Ω source impedance. In our case, the function generator connects to a transistor whose gate‐to‐source resistance is much higher. As a result, the transistor gate acts as a circuit break, leading to a doubling of the voltage at the gate. Therefore, in the examples shown in Figure 2c, the function generator is nominally configured to output 4 V voltage pulses, which however results in an 8 V bias output at the transistor gate.

The thermal response of SOI heater platform to voltage pulses was modeled via the finite element method (FEM) using the COMSOL Multiphysics package. In the COMSOL model, experimentally assessed temperature‐dependent doped Si conductivity (Figure S1, Supporting Information) and PCM thermal properties^[^ ^69^ ^]^ were used. Other material parameters were quoted from the COMSOL Multiphysics v6.1 database.^[^ ^70^ ^]^ In the simulations, a transient model was used to model amorphization while a steady‐state model was employed for crystallization, since the crystallization pulse is much longer than the thermal time constant of the heater. For amorphization, a top‐hat 32 V amplitude and 12 μs duration voltage pulse were applied across the heater. For steady‐state simulations, a constant 17 V bias was applied. The system started at 298 K and a constant temperature boundary condition was enforced to the bottom Si substrate. A convection heat flux was applied to the top surface using a heat transfer coefficient of 20 W m^−2^ K^−1^,^[^ ^71^ ^]^ although our results indicate that the convective heat removal is negligible in comparison to conductive heat dissipation through the substrate.

The thermal simulation results are plotted in Figure 1d, showing the temperature as a function of time during amorphization, and in Figure 1e mapping the steady‐state temperature profile across the heater during crystallization. Simulation results for 200 and 100 μm‐sized square heaters on 3 μm of buried oxide (BOX) along with results for a 100 μm‐sized heater on 1 μm BOX are provided. The voltages applied were chosen so that the maximum temperatures at the center of the heaters are nearly identical. The 200 and 100 μm‐sized heaters with 3 μm BOX exhibit similar temperature ramp and decay characteristics, whereas the heater on 1 μm BOX shows a much faster temperature transient response. The observation implies that heat removal occurs mostly through the substrate, and in‐plane heat dissipation is negligible. The fitted thermal decay time constant for the 100 and 200 μm heater on 3 μm BOX is 7.2 and 7.1 μs, respectively, and 1.9 μs for the 100 μm heater on 1 μm of oxide. Characterization of PCMs with rapid crystallization kinetics can thus be performed using SOI wafers with thin BOX.

The PCM‐integrated microheater arrays can be monitored in situ using noncontact optical techniques such as optical microscopy, micro‐Fourier transform infrared (micro‐FTIR) spectroscopy, and micro‐Raman spectroscopy. These techniques can be used to extract an extensive set of information including PCM morphology, phase composition, local temperature, optical constants, phase transition kinetics, and endurance, as we will discuss later. The devices can also be analyzed ex situ using destructive methods such as transmission electron microscopy (TEM) to reveal spatially resolved PCM composition and structural information, which proved a powerful tool to understand failure mechanisms of PCMs (detailed analysis to be published in a separate article). For optical imaging, an AmScope AF205 autofocus camera was used for image acquisition. For white balance, the aluminum pads were used with resulting sensitivity values of R 15, G 32, and B 68. A Thermo Scientific Nicolet iS50 spectrometer with a microscope using a 15*X* 0.58 NA ∞/*V* Reflachromat condenser and an HgCdTe detector was used for transmission micro‐FTIR. An inVia Renishaw spectrometer using a 50× objective (NA = 0.5) and optical resolution 0.96 μm was used for Raman spectroscopy measurements. The laser for Raman spectroscopy operates at 785 nm. In Raman measurements, it is important to ensure that the laser excitation power is sufficiently small to prevent optically induced structural changes.

## 3 Multimodal PCM Characterization

**Figure 3:**
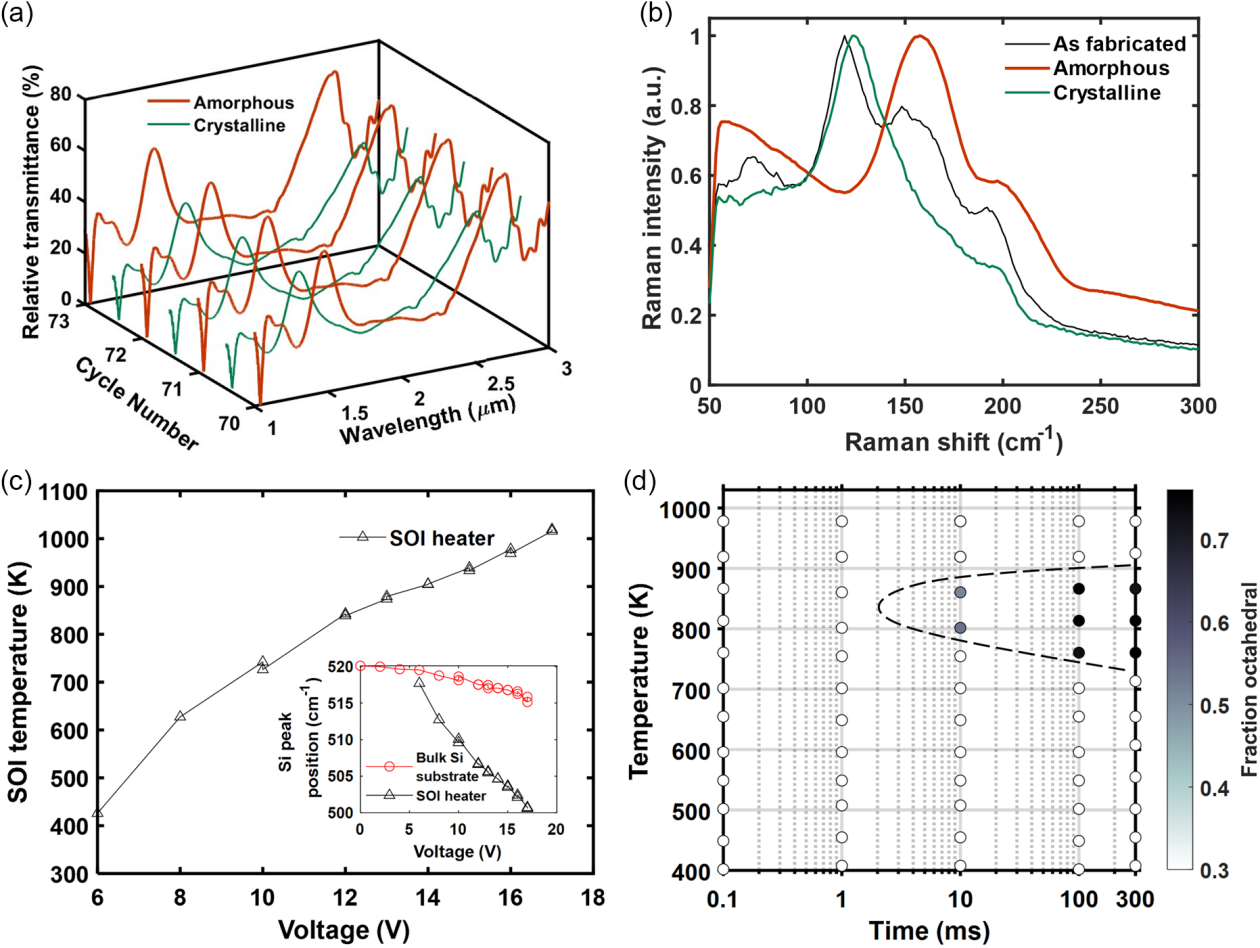
a) Transmittance of a PCM‐integrated SOI heater measured in situ via FTIR. b) Raman spectra of the PCM (GSST) from an as‐fabricated device (black curve), after recrystallization (green curve), and after reamorphization (red curve). c) SOI heater temperature measured using Raman spectroscopy as a function of applied voltage at steady state. The inset shows the Si Raman peak positions for the SOI heater and bulk Si substrate vs applied voltage across the heater. d) Measured TTT diagram of GSST (data from Vitale et al.^[^ ^72^ ^]^). The dashed line encircles the crystallization region.

Here, we take a GSST‐integrated heater array to illustrate the repertoire of in situ analysis capabilities empowered by the platform. We first use transmissive FTIR to characterize the transmittance of patterned PCM structures. **Figure** **3**a presents the FTIR spectra of the GSST‐integrated heater over multiple cycles showing reversible transmission features. The background for the relative transmission was a silica microslide and the sample was secured with Norland optical adhesive 60 to the microslide. The illumination was from the backside, starting with the SiO_2_ microslide. Because of the silica‐to‐air interface and the background used, the relative transmission of the patterned GSST is higher than if the background was air.

The phase composition of the PCM is then quantified via Raman spectroscopy. Figure 3b shows the Raman spectra collected on the device. The peak at 120 cm^−1^ is primarily linked to vibrations of the Ge‐6Se octahedra characteristic of ordered crystalline phases, and the crystalline phase fraction can be calculated by fitting the Raman spectra with Raman active vibrational modes of GSST.^[^ ^72^ ^]^ From Figure 3b, we also see that GSST in the as‐fabricated device is partially crystallized, evidenced by the appearance of the Ge–6Se octahedra peak and likely caused by several heat treatment steps involved in the fabrication process.

In addition to determining the phase structures of PCM, Raman spectroscopy also enables noncontact, self‐calibrating, and in‐line probing of local temperatures at the SOI heater surface with micrometer‐scale lateral resolution and ±10 °C accuracy. This is accomplished by tracking the temperature‐dependent spectral shift of the single crystal Si Raman peak.^[^ ^73^ ^]^ In our experiment, we observed a clear redshift and subsequent splitting of the Si Raman peak into two as the applied DC voltage increases (Figure 3c inset). At low voltages, Raman peaks from the SOI heater and the Si substrate coincide due to a negligible heating effect. At higher voltages, the redshift of the SOI Raman peak is used to extract the heater temperatures plotted in Figure 3c, which also agrees well with our FEM simulations. We note that while here we use standard Raman microscopy to measure steady‐state temperatures at the heater, it is also possible to leverage ultrafast Raman thermometry to quantify the transient temperature evolution at the sub‐nanosecond time scale.^[^ ^74^ ^]^

Combining the phase composition and temperature information enables quantitative evaluation of the time‐temperature‐transformation (TTT) diagram in PCMs, which is of vital importance not only to the understanding of phase transformation kinetics but also to PCM switching process optimization.^[^ ^72^ ^]^ Specifically, high‐throughput isothermal PCM crystallization experiments can be performed on the microheater array. The crystallization temperatures are calibrated using Raman during crystallization and the phase composition is assessed via Raman fitting after crystallization. As an example, Figure 3d shows a measured TTT diagram of GSST. In the diagram, each point corresponds to one isothermal crystallization experiment on a microheater for a given heat treatment temperature and duration. The diagram points to a nose temperature of ≈820 K, implying that crystallization of GSST should ideally be performed at this temperature to expedite the transition. The TTT diagram can be further refined by adding more data points to yield more accurate kinetics, an advantage afforded by the high‐throughput testing capability on our platform.

The cycling behavior of the PCM is investigated through optical microscopy. We have set up an automated collection system and an image processing algorithm (available through Github^[^ ^68^ ^]^). The system records microscopy images of the sample across three color channels (red R, green G, and blue B) of the camera, performs background subtraction (filtering out the non‐PCM covered areas by setting a contrast threshold), normalizes the brightness level, and outputs the optical contrast evolution over cycles. The optical contrast is characterized by the differential mean analysis (DMA) parameter $\Delta \text{DMA}\;=\;{\left[\frac{\bar{I_{\text{PCM}}}}{I_{\text{Bckg}}}\right]}_{\text{Cr}}-{\left[\frac{\bar{I_{\text{PCM}}}}{I_{\text{Bckg}}}\right]}_{\text{Am}}$. Here, *I* represents the optical intensity in one of the color channels, the subscripts PCM and Bckg denote the intensity from the PCM film and the background (non‐PCM covered region), respectively, and the subscripts Cr and Am refer to quantities associated with crystalline and amorphous states, respectively. The evolution of DMA over cycles provides a direct measure of the endurance as well as consistency of the PCM cycling process. Because the recorded pixel intensity is dependent on the light source spectrum, camera pixel spectral sensitivity, color filter characteristic, gain applied, and also potential image processing such as white balance or image sharpening, the ΔDMA value is only a semiquantitative estimation of the optical contrast of the sample. If a region of interest is set, the performance of various structures can be analyzed separately.

**Figure 4:**
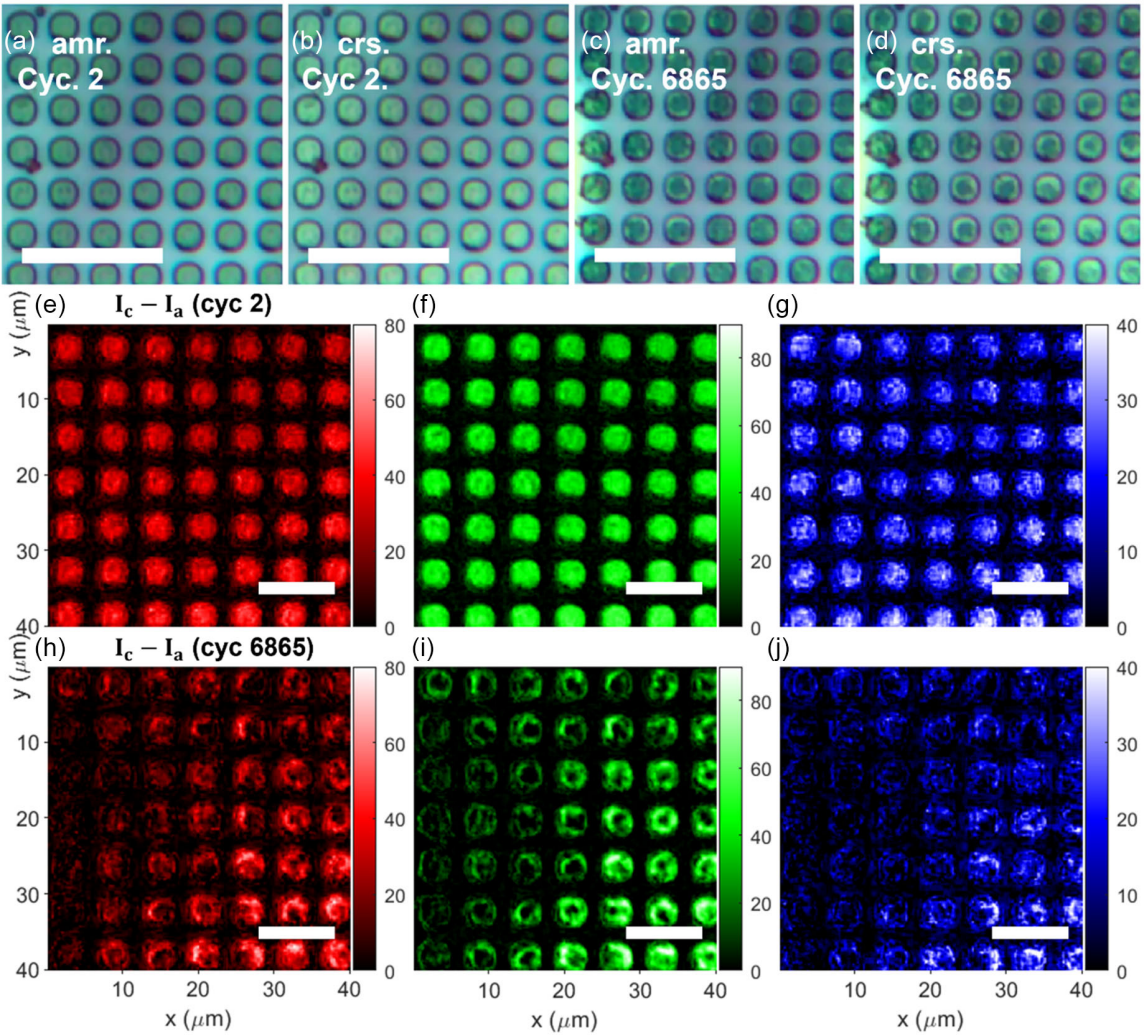
a–d) Optical microscopy images of a GSST‐integrated heater in amorphous (a,c) and crystalline (b,d) states. (a,b) show uniform switching from the initial cycles of the sample and (c,d) imply nonuniform switching due to partial failure after 6865 switching cycles of the device. Scale bars: 20 μm for (a–d) and 10 μm for (e–j). e–j) Pixel intensity differences between the crystalline and amorphous state across the R, G, and B color channels between images (a,b), (e–g), and images (c,d) (h–j), respectively.

**Figure 5:**
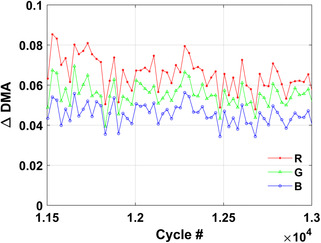
DMA parameter evolution over cycles in a GSST device with 180 nm thick GSST and 740 nm thick SiN_*x*_ encapsulation.

As an example, **Figure** **4** presents data collected from two samples where the PCM films are patterned into cylinder arrays. Figure 4a,b shows spatially uniform switching of PCM evidenced by the contrast maps in Figure 4e–g. In comparison, Figure 4c,d,h–j shows data collected on a sample that has undergone 6865 switching cycles and has partially failed, as only selected PCM unit cells (at the bottom right corners of the images) continued to switch. The device can then be sectioned with a focused ion beam and analyzed with TEM. Differences in microstructures and composition gradients between the “active” and “dead” unit cells provide valuable insights elucidating the mechanisms leading to PCM failure (the details of which will be summarized in a forthcoming publication). **Figure** **5** shows the measured DMA on a sample designed with enhanced reliability, indicating consistent switching over more than 10 000 cycles.

## 4 Conclusion

We have presented a multifunctional platform capable of characterizing the structural, optical, kinetic, thermal, and cycling behavior of optical PCMs. The platform is based on SOI microheater arrays and an electrothermal switching scheme representative of the PCM deployment environment when integrated with industry‐standard silicon photonic devices. We demonstrate that the platform enables in situ, quantitative characterizations of the phase composition, optical constants, temperature profiles, TTT diagram, cycling endurance, and material uniformity of PCM thin films. It can also be coupled with combinatorial deposition to facilitate high‐throughput screening of new PCM compositions, or combined with ex situ characterization techniques such as electron microscopy to elucidate the microstructural evolution and failure mechanisms of PCMs. The microheater array devices can be fabricated leveraging commercially available photonic foundry services, and we open‐sourced hardware and software designs of the characterization platform, allowing the broader PCM community to make use of its unique capabilities. It is our hope that the work will expand and expedite the discovery and qualification of new optical PCMs for diverse photonic applications.

## Conflict of Interest

The authors declare no conflict of interest.

## Supporting information

Supplementary Material

## Data Availability

The data that support the findings of this study are openly available on GitHub and Zenodo at https://doi.org/10.5281/zenodo.8076261, reference number 8076261.
