# Supplementary material for: An Open‐Source Multifunctional Testing Platform for Optical Phase Change Materials
Source: Small Sci. 2023 Nov 20;3(12):2300098. doi: 10.1002/smsc.202300098 (PMC11936044; doi:10.1002/smsc.202300098)
Supplement: Supplementary file 1 — Supplementary Material [file SMSC-3-2300098-s001.pdf]

Supporting Information

Title An Open-Source Multi-functional Testing Platform for Optical Phase Change Materials

*Cosmin-Constantin Popescu, Khoi Phuong Dao, Luigi Ranno, Brian Mills†, Louis Martin, Yifei Zhang, David Bono. Brian Neltner, Tian Gu, Juejun Hu**

Department of Materials Science & Engineering, Massachusetts Institute of Technology Cambridge, MA 02139, USA

E-mail: hujuejun@mit.edu

† Draper Scholar, The Charles Stark Draper Laboratory, 555 Technology Square, Cambridge, MA 02139, USA

*Kiumars Aryana, William M. Humphreys, Hyun Jung Kim**

NASA Langley Research Center, Hampton, VA 23681, USA

E-mail: hyunjung.kim@nasa.gov

Steven Vitale, Paul Miller, Christopher Roberts

Advanced Materials and Microsystems Group, MIT Lincoln Laboratory Lexington, MA 02421, USA

Sarah Geiger, Dennis Callahan, Michael Moebius

The Charles Stark Draper Laboratory, Inc., Cambridge, MA 02139, USA

Myungkoo Kang, Kathleen Richardson

CREOL, The College of Optics & Photonics University of Central Florida Orlando, FL 32816, USA

Carlos A. Ríos Ocampo

Department of Materials Science & Engineering University of Maryland College Park, MD 20724, USA

DISTRIBUTION STATEMENT A. Approved for public release. Distribution is unlimited.

This material is based upon work supported by the Under Secretary of Defense for Research and Engineering under Air Force Contract No. FA8702-15-D-0001. Any opinions, findings, conclusions or recommendations expressed in this material are those of the author(s) and do not necessarily reflect the views of the Under Secretary of Defense for Research and Engineering.

The conductivity of doped silicon was estimated starting with a device resistance of 44 Ω at room temperature and assuming that the resistance corresponded to the doped heater region solely. The voltage and current reported by the voltage source were recorded and the corresponding resistance computed. For the conductivity, a 150 µm square doped Si heater with 116 nm SOI thickness was used.

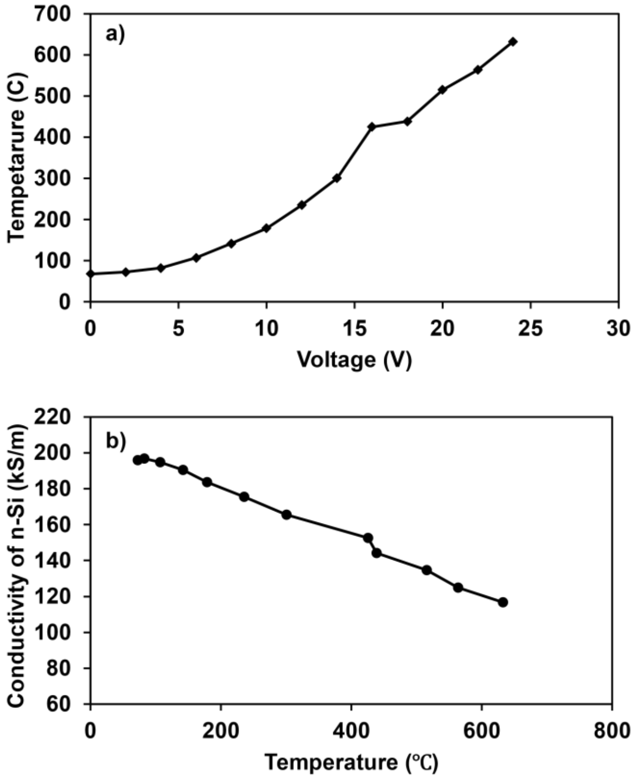

Figure S1 a) Temperature estimated from Raman spectroscopy measurements vs. voltage and b) calculated doped silicon conductivity vs. temperature
